# Psychological and ethical issues raised by genomic in paediatric care pathway, a qualitative analysis with parents and childhood cancer patients

**DOI:** 10.1038/s41431-024-01653-4

**Published:** 2024-07-13

**Authors:** Marion Droin-Mollard, Sandrine de Montgolfier, Anne-Paule Gimenez-Roqueplo, Cécile Flahault, Arnaud Petit, Franck Bourdeaut, Sophie Julia, Emmanuelle Rial-Sebbag, Isabelle Coupier, Fatoumata Simaga, Laurence Brugières, Léa Guerrini-Rousseau, Béatrice Claret, Hélène Cavé, Marion Strullu, Lucile Hervouet, Khadija Lahlou-Laforêt

**Affiliations:** 1 UF of Psychology and Liaison and Emergency Psychiatry, DMU Psychiatry and Addictology, Assistance Publique Hôpitaux de Paris, Hôpital Européen Georges Pompidou, F-75015 Paris, France; 2 IRIS Institut de Recherche Interdisciplinaire sur les Enjeux Sociaux (UMR 8156 CNRS—997 INSERM—EHESS—UPSN), Campus Condorcet, Aubervilliers, France; 3 University of Paris Est Créteil, Créteil, France; 4 Aix Marseille Universite, Inserm, IRD, SESSTIM, Sciences Economiques & Sociales de la Santé & Traitement de l’Information Médicale, ISSPAM, Marseille, France; 5 Département de Médecine Génomique des Tumeurs et des Cancers, Consultation d’oncogénétique Multidisciplinaire des Cancers Rares, Assistance Publique Hôpitaux de Paris, Hôpital Européen Georges Pompidou, Paris, France; 6 Université Paris Cité, PARCC, INSERM, Paris, France; 7 Université Paris Cité, Laboratoire de Psychopathologie et Processus de Santé UR4057, Paris, France; 8 Service d’Hématologie et d’Oncologie Pédiatrique, Hôpital Armand Trousseau, APHP, Sorbonne Université, Paris, France; 9 SIREDO Pediatric Oncology Center, Laboratory of Translational Research in Pediatric Oncology—INSERMU830, Institut Curie, Paris Sciences Lettres Research University, Paris, France; 10 Université Paris-Cité, Paris, France; 11 UMR 1027 INSERM, University of Toulouse & Toulouse University Hospital, Toulouse, France; 12 Medical Genetics Department, Purpan Hospital, Toulouse, France; 13 UMR 1027 INSERM, University of Toulouse & University Toulouse III—Paul Sabatier, Toulouse, France; 14 CHU Montpellier, Hôpital Arnaud de Villeneuve Montpellier, Service de Génétique Médicale et Oncogénétique, Montpellier, France; 15 INSERM896, CRCM Val d’Aurelle, Montpellier, France; 16 Department of Genetics, Institut Curie, Paris, France; 17 Department of Pediatric and Adolescent Oncology, Gustave Roussy Cancer Campus, Université Paris-Saclay, Villejuif, France; 18 Molecular Predictors and New Targets in Oncology, Inserm U981 Team “Genomics and Oncogenesis of Pediatric Brain Tumors”, Gustave Roussy Cancer Campus, Université Paris-Saclay, Villejuif, France; 19 Psycho-Oncology Unit, Supportive Care Department, Gustave Roussy Cancer Campus, Université Paris-Saclay, Villejuif, France; 20 Assistance Publique des Hôpitaux de Paris (AP-HP), Hôpital Robert Debré, Département de Génétique, Paris, France; 21 INSERM UMR_S1131, Institut de Recherche Saint-Louis, Université Paris-Cité, Paris, France; 22 Assistance Publique des Hôpitaux de Paris (AP-HP), Hôpital Robert Debré, Service d’Hémato-Immunologie Pédiatrique, Paris, France

**Keywords:** Paediatrics, Psychology, Ethics

## Abstract

In paediatric oncology, genomics raises new ethical, legal and psychological issues, as somatic and constitutional situations intersect throughout the care pathway. The discovery of potential predisposition in this context is sometimes carried out outside the usual framework. This article focuses on the views of children, adolescents, and young adults (AYA) with cancer and their parents about their experience with genomic testing. Forty-eight semi-structured interviews were performed with children or AYAs with cancer and one of their parents, before and/or after receiving the genetic test results. The interviews were fully transcribed, coded and thematically analysed using an inductive method. This analysis revealed several themes that are key issues: perceived understanding and consenting, apprehension about the test outcomes (expectations and fears), perception and attitude towards incidental findings. The main expectation was an aetiological explanation. Children and AYAs also emphasised the altruistic meaning of genetic testing, while parents seemed to expect a therapeutic and preventive approach for their child and the rest of the family. Parents were more concerned about a family risk, while patients were more afraid of cancer relapse or transmission to their descendants. Both groups suggested possible feelings of guilt concerning family transmission and imaginary representations of what genomics may allow. Incidental findings were not understood by patients, while some parents perceived the related issues and hesitated between wanting or not to know. A multidisciplinary approach would be an interesting way to help parents and children and AYAs to better grasp the complexity of genetic and/or genomic testing.

## Introduction

In paediatric oncology, germline or somatic genome sequencing is proposed to characterise the cancer type, personalise therapy, obtain data on germline variants for preventive and familial implications, and for clinical research purposes. Thus, patients and their parents are confronted with situations where somatic or germline tests, targeted gene sequencing or genome sequencing intersect. Genome sequencing brings eventually information on constitutional variants linked to the current disease, but also additional data, for instance pathogenic variants not directly related to the initial indication (incidental findings or secondary findings), and variants of unknown significance apart from a usual consultation on genetic predisposition in patients [1–3]. Whatever the pathology, genetic testing is associated with psychological and ethical issues for the child, parents, and professionals [4–8]. Guidelines for genetic testing in adults have been published [9–11] and they should be adapted to children, adolescents, and young adults (AYAs) [5, 12, 13]. Studies on ethical and psychological issues associated with genetic and genomic testing in children and AYAs with cancer are limited. In a narrative review of 18 articles, we explored the perspectives of parents and to a lesser extent of children and AYAs with cancer and highlighted areas of ambivalence concerning the subjective implications of those tests (desire for treatment, desire for knowledge, uncertainty, and guilt) [14]. The aim of this qualitative study was, thanks to a suitable methodology, to understand how parents and also children and AYAs perceive genetic or genomic testing and to analyse their psychological implications, expectations, and representations before and after the result announcement, whatever the type of test proposed.

## Methods

### Context

GeneInfoKids is a national project financed by the French National Cancer Institute with three axes: ethical, legal and psychological issues raised by next generation sequencing for children and AYAs with cancer. The main objective of the psychological axis is to describe the psychological implications in families of patients undergoing genome sequencing. It includes a qualitative study (described in this article) and a quantitative study based on the themes defined by this qualitative study.

### Participant recruitment

The inclusion criteria were children, adolescents and young adults (AYAs), also referred to as patients in the text, with cancer or past history of cancer age between 10 and 25 year at the time of inclusion in the study, having undergone somatic or constitutional testing in a clinical or research context (i.e. MAPPYACT) at one partner centre (Gustave Roussy, Hôpital Robert-Debré, Hôpital Armand-Trousseau, Institut Curie), and French speaking [15, 16]. Children and AYAs who met the inclusion criteria and at least one of their parents received an information leaflet by the clinical team. Then, a research psychologist contacted by phone the parents (for minors) or the patients directly (for adults), explained again the study, and validated their consent to participate.

### Interviews

Interview guides were developed in a vocabulary and style suitable for parents and children and AYAs, based on literature data and on the clinical experience of the study investigators involved in genetic testing for childhood cancer [17] (supplementary materials). Two semi-structured interviews were planned: one after the genetic test proposition and one after the genetic test result announcement. In the first interview, the main discussion topics were: family and disease context, interest in genetic or genomic testing, knowledge of the possible result types (e.g. primary result, incidental findings), expectations and fears, consent decision-making modalities. In the second interview, the main topics were: knowledge about the possible results, associated emotions, match between expectations and results, consequences and implications of the results, temporality of genetic testing in the care pathway, need of a dedicated psychologist consultation. Only parents were asked to give their opinion on their preparation, from information to announcement. Information on the pathology and type of test performed were also collected.

### Data analysis

All interviews were audio-recorded, transcribed and pseudonymized. They were analysed using MAXQDA 2020 with a systematic coding and thematic analysis using an inductive method [18]. Eight interviews were double coded to define the themes, the others were coded by one researcher and discussed with the other one. The final thematic analysis plan was discussed by three researchers. The number of occurrences corresponds to the number of patients or parents who stated these ideas which were questioned or not according to the clinical context and understanding.

## Results

### Description of the semi-structured interviews

**Fig. 1:**
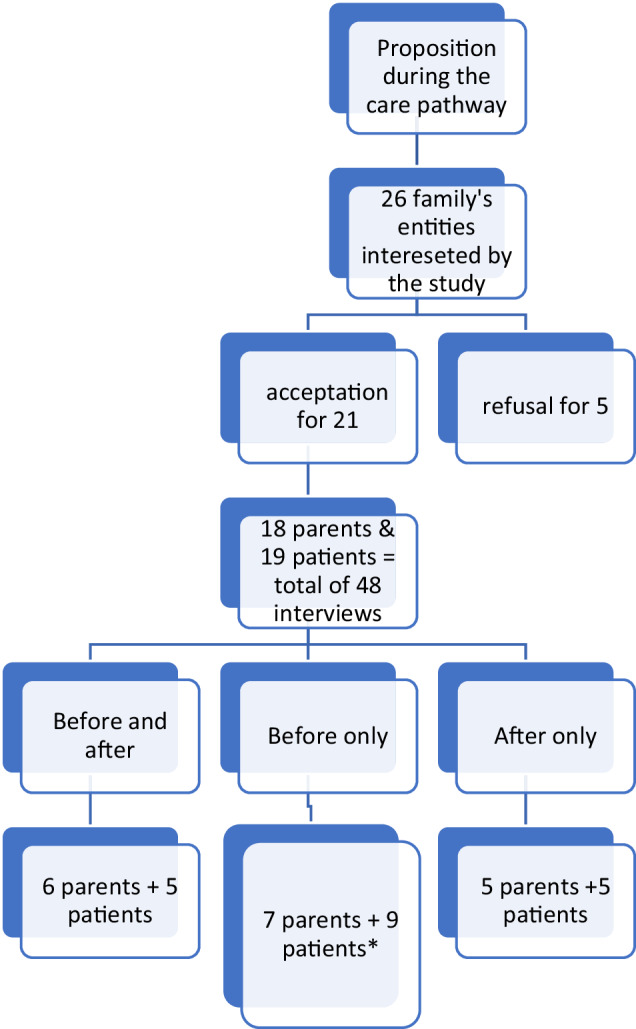
Interviews presentation. *Deterioration of the child’s condition or child at the end of life or inclusion terminated before the result is delivered.

Interviews (*n* = 48) took place between July 2020 and May 2021. They lasted 15–90 min (mean: 35 min): 29 interviews (60%) were face-to-face in the hospital and 19 (40%) were by videoconference, due to the COVID-19 pandemic. In total, 21 families (19 children and AYAs and 18 parents) were interviewed before and after, only before, or only after the test (Fig. 1 and Table 1). Both parents had the opportunity to participate, but in only one family both parents were interviewed due to their availability. Among the families who agreed to be contacted for this study, five declined to participate (refusal by one parent) mainly because they felt overwhelmed or insufficiently informed on the genetic approach (confirmation rate: 80.8%). Therefore, we interviewed 14 patients with cancer and 13 parents before the test disclosure and 10 patients and 11 parents afterwards. The thematic analysis of the interviews led to the construction of four categories: children and AYAs, parents, before, and after the genetic results (Tables 2 and 3).

**Table 1:** Characteristics of persons interviewed and timing of the interviews.

Variable	Children and AYAs (*n* = 19)	Parents (*n* = 18)
Gender		
Male	8	5
Female	11	13
Timing of the interviews		
Before the genetic test results	14	13
Before genetic test only	9	7
After the genetic test results	10	11
After genetic test only	5	5

**Table 2:**
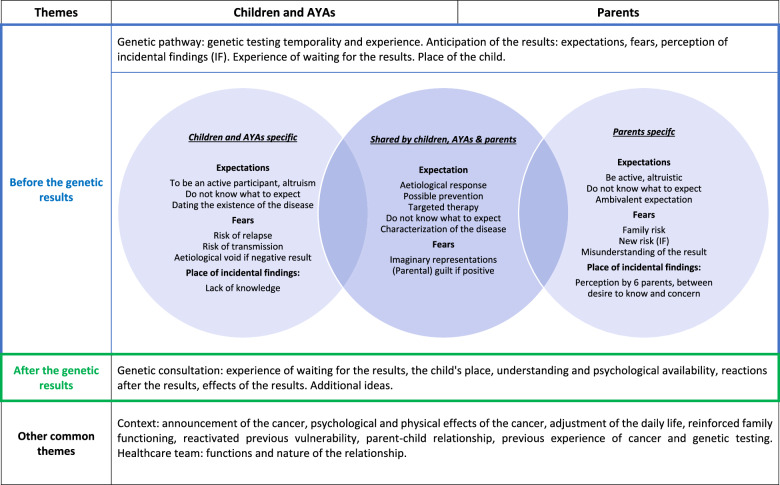
Major themes and subthemes.

**Table 3:** Themes and quotations (names have been changed).

Themes and quotations: Factors influencing genetic testing decision-making	Participants
Antagonistic feelings between (or: attitudes to) genetics and cancer	
*"I know that we are all against time and against the clock that I don’t want to be the only obstacle and then regret it later." [...] "An aggressive disease, it doesn’t give you the choice to decide anything. [...] So what if we make a decision, if we agree on the..., on the steps. We’re not going to torture ourselves"*	Sara’s mother
*"The aim was for her to live as well as possible, and the others too, to enjoy what we have and then we’ll see. So, I must admit that genetic testing was the least of my worries."*	Désirée’s mother
*"I didn’t care much either, well no, but um... whether it was genetic or not, it wasn’t going to affect what is, well a little bit, but it wasn’t going to affect at the time, but maybe later on it would have affected me."*	Lucien, 13 years old
Psychological availability and understanding	
*"In any case, when I get bad information, I tend to... [...] put myself in automatic mode. [...] The emotions are not there at all. I feel absolutely nothing. I feel like I’m in a parallel world. And so there I am, I’m in automatic mode, it’s going to be bang, bang, bang, bang, the way the thing goes. [...] So, maybe it was the same thing with the genetic test. I took the genetic test, that’s it. But afterwards, the rest..."*	Adam’s mother
*"We knew that there were tests underway but we weren’t more interested in what was being done because for us it was... a bit too much information. And so um... we were happy with what we were... given as information and when we needed more we asked questions. But uh... no too much, too much knowledge I think it’s not... it’s not necessarily what would have suited us."*	Tom’s mother
*"There are things I do not have... information I do not have, because I’m quite depressed [...] I was so depressed at the time that even if I had been informed, it wouldn’t have helped. I wouldn’t have understood..."*	Tom, 15 years old
*"In fact, above all, we hear the good things, and the complicated things... yeah, we’ve already got quite a few of those, we’re not going to hear them too much either."*	Ulysse, 16 years old
*"It was already a big burden to learn about the tumour. So after that, the other, well, the other information over and over again, it was a bit heavy."*	Cerise, 15 years old
*"I didn’t really, really, really understand the process. Because I... well, I don’t know if we were really asked our opinion at... Well, I think it slipped my mind a little bit at one point in fact."*	Lucien’s mother
*"I think they made me sign a paper relevant to that. We’re not really able to sign anything, well, in full consciousness, I think. We’re in a bit of a dead point, we don’t really know what’s happening to us, we’re digesting every piece of information without really digesting it."*	Désirée’s mother
*"We didn’t even take time to think about it. It was ’Yes’ for both of us, right away."*	Léa’s mother
*"Well, it’s too hard to understand"*.	Lucien, 13 years old
*"I don’t even know if I’ve had it done, because this morning I had a blood test, I don’t know if that’s why."*	Tara, 18 years old
*"I mean, how is genetic testing done? If they tell me how it’s done, maybe I’ll remember if I’ve had one or not. [...] I don’t really know, I admit I don’t really know. I took a test for... sperm test, I think... "*	Ulysse, 16 years old
*"When I think about it, my head was down. I mean, during my appointment, I was only thinking about that and not about other things. So, it allows you to... understand the meaning…"*	Théophile, 16 years old
*"How it’s going to work, or what the treatment is, is this the first time they’re going to use it? Is my daughter going to be like, I don’t know, a, a test subject? I didn’t ask these questions and I don’t want to ask them. I’m assuming they’re also doing their best to find a cure for Sara. That’s all. I see it like that, I don’t want to see it any other way, that it’s going to be (laughs), that it might traumatize my conscience (laughs)."*	Sara’s mother
Influence of the consultation type	
*"We don’t think, [...] we don’t have the distance at certain times, here during an appointment like this with the doctor, he throws a lot of information at us about radiotherapy, well, things that we discover, well, we take in a lot of things at once, here we can’t, I can’t manage to take a step back and to... I haven’t managed to ask questions about other, um... about this subject"*	Lucien’s mother
*"I even had the impression that he [the oncologist] wasn’t going to talk about it [genetics] in the end because it really came up at the end of the discussion and there wasn’t much time to spend on it, I think."*	Lucien’s father
*"There was this question of genetic predisposition, and then Professor XX wanted Cerise to take part in a clinical trial, so we didn’t even have time to discuss it with Cerise, i.e. we were told the protocol straight away and in fact our questions were all about that protocol. So in the end, it almost... erased, um, all the questions that had been asked about genetic predisposition, you see, even Cerise remained, I think, mentally much more so after the protocol that was announced to her than... everything that was linked to genetics."*	Cerise’s mother
Somatic/germline testing, a blurred perception by families	
*"I say to myself that if it’s for the good of Chloé, to really know how to target, I say to myself, I might as well go [...] So that it’s not so heavy..."*	Chloé’s mother
*"What interested me was the subtype and knowing what the risk really was. Was it a standard risk, a high risk? Would it have an impact on my son’s treatment in the immediate future? We’re short termists, that’s for sure."*	Lucien’s father
*"I agreed to take part in the research, and so it was as if I had been told... well, that as I had taken part in the research, I had to do a genetic test [...] Finally, the research that we did, that we started at the beginning, which took place at XX, I think, from time to time, they need blood tubes to... they take an extra tube of blood to analyse. A bit like that"*	Yvan, 14 years old
Themes and quotations: Is there room for an informed decision?	Participants
Intricacies of the motivations of professionals and/or parents and their child	
*"She told us that she was in fact one of the doctors who were oncogenetic specialists and therefore... That potentially, we would have to do tests because of our daughter’s age and the type of cancer she had. So that’s it. That’s how she asked us if we agreed. So my husband and I... we immediately... [...] wanted to see if, in relation to the samples we had, it came from our own genes, and if we could potentially pass it on to her sister as well, and then to see if we needed to carry out more extensive research for the whole family as well, because it was the fact that she had adult cancer in fact."*	Léa’s mother
*"They wanted to see if, for example, in this one they could find something that could explain the disease or something that linked all the cases of this disease."*	Yvan, 14 years old
*"It was my parents who wanted to do it."*	Adam, 12 years old
Decision-making feelings	
*"I mean, I haven’t really heard much about it. I mean I’ve... yes, I have, but I haven’t... I haven’t really noticed it."*	Yvan, 14 years old
*"I didn’t think much of it"*.	Adam, 12 years old
*"We have to start it as soon as possible [...] the reactivity that you have had has been good and well, we have a cancer that is just localized at the level of the blood cells. So we mustn’t waste any time."*	Aïnoa’s father
*"There were more tubes, it took a little longer to take the sample. But in terms of the stress he has when he takes blood samples, it didn’t add an extra test. So there, we didn’t even hesitate."*	Adam’s mother
*"I don’t know if we were really asked our opinion at... Well, I think it slipped my mind a bit at one point actually."*	Lucien’s mother
Themes and quotations: The child*’*s place	Participants
*"I wanted it to end in fact, I was tired and everything, and in fact I forgot everything about that time, the questions, so I didn’t ask at all, not even the questions I wanted to ask "*	Lucien, 13 years old
*"The approach they take with the children too, because it’s simple words, well at least the doctors who... It was very simple words. And then they give them time to think and they come back to them and say: "Have you understood everything? Do you want me to explain it again? That’s it. We feel that they are very close to the children in this respect."*	Léa’s mother
*"We were supervised by a doctor who was very... well, adorable, well, a very gentle way of dealing with Tom. And she spoke to him in very simple words. And... she let him talk a lot and rephrase what she was saying to make sure he understood."*	Tom’s mother
*"In fact, we quickly moved on, so for her I think it was important and I realise that we didn’t really discuss it again afterwards, saying, "Here we go again", because in the end, it’s like an expert talking about a subject and he thinks you’re clear on it, but in fact, no, not completely."*	Cerise’s mother
Themes and quotations: Anticipating and reacting to the genetic test outcome	Participants
Expectations	
*"It is being in the dark that is difficult, not knowing. And having the answers to the questions allows you to build."*	Capucine, 24 years old
*"There is always the hope of finding"*	Adam’s mother
*"We don’t want anyone in our family to go through what we went through for a year and a half. [...] I say to myself, it’s our responsibility and afterwards it’s a family discussion. [...] for our children, and our nephews and nieces, we didn’t... it was immediately when we were told about it, it was... I mean we didn’t even take time to think about it."*	Léa’s mother
*"I’m in total confusion about this. I have no idea what they can tell us. [...] I don’t know if it would do anything for him. In the same way that, in fact, I don’t even know if it will bring me anything."*	Désirée’s mother
*"To determine in the end the... I don’t know how to put it, the identity card in fact of the, of the bad cell in order to be able to put in place the best procedure to fight it."*	Aïnoa’s father
Fears	
*"I didn’t want to hear them say that it was possible to be cured and then just go back into a longer phase of the disease."*	Sophia, 13 years old
*"I’m not going to blame Mum or Dad, because they didn’t do it on purpose. [...] Some people might blame them if it was their parents, but I won’t blame them."*	Léa, 10 years old
*"I would prefer it if it wasn’t that at all and that the rest of the family was protected, not affected by this genetic thing and this... cancer. Afterwards, it can happen for other reasons. But we shouldn’t be told ah well, ah well it’s certain that his sister, his brother will have such and such a disease, such and such a cancer. I think it’s bad enough as it is, there’s no need to add stress and worries*.	Lucien’s mother
*"Well, of course, it’s guilt because I say to myself, well, this is... well, we made the children, and it’s our genes that got mixed up."*	Léa’s mother
Themes and quotations: Reactions to the genetic test outcome	Participants
Emotions	
*"It was a bit difficult to... to discover that... it wasn’t hereditary. Because then we don’t have an answer."*	Cerise, 15 years old
*"I’m happy anyway, a little bit anyway, but I don’t know, I’d like to know where it comes from."*	Lucien, 13 years old
*"I had the impression of being left wanting, of not, of not having uh, more uh... See, we don’t have any more details than that in fact, yes, so, we’re still a bit uh, in fact we’re so waiting for an answer that when we’re, when we arrive at the appointment and then she tells us that... "*	Théophile’s mother
*"It only reinforces an uncertainty"*.	Cerise’s mother
Effects of the results	
*"It won’t be because of dad or because of mum or I don’t know, or if we have to do one of the more in-depth analyses. Well, that’s the way it is. But in any case, Cerise was born from a union of two people who loved each other and she was conceived in love. And that’s what we remember."*	Cerise’s mother
*"Now we laugh about it, even though we know it, that I’m their... their son, er... And that they are my sisters. Now we laugh about it because we’re really sure and certain."*	Tom, 15 years old
*"I think it was really the genetic test that renewed the uh... the question of... where the disease came from."*	Cerise, 15 years old
*"At no time did I have any doubt about it. At no time did I say to myself: "No, it’s hereditary". No, because for me, the cause is external, and I am not the cause. So, obviously, it’s not genetic, it’s not hereditary, well, it’s maybe... it’s a genetic disorder that occurred, but not due to hereditary causes"*	Morgan’s father
Themes and quotations: Perception of incidental findings	Participants
*"I say to myself that necessarily, with analyses of this kind, there are other things that can emerge of course. There may have been other predispositions to something else, perhaps just by analysing his tumour. "*	Cerise’s mother
*"I would say that it’s possible that we’ll realise that, well for example, Lucien or one of his brothers and sisters may be more susceptible to, yes, autoimmune diseases, or that sort of thing and uh that will tell us, well uh, in a few years, it may happen that... [- C: Diseases different from cancer? -] Exactly... or cancer too... "*	Lucien’s father
*"Well, my fear is that, through what she has already had as an illness, more serious things will be discovered. That’s more my fear. (...) Another tumour, another hidden disease."*	Eléonore’s mother
*"We may be able to detect genetic abnormalities that may point to something other than the pathology we are currently treating. [...] it’s worrying, if of course, if you tell me ah there’s a huge genetic anomaly, obviously, it’s going to be worrying. But for the moment, no it’s not, and for the moment I see it as a source of knowledge, of insight and I think it’s better to know than not to know."*	Lucien’s father
*"It may be scary, but not as much as that, because we are into prevention and I like to be warned, or to take precautions before reliving what I experienced with Sara. Even if, um, it’s annoying, but it’s better, because if there’s already a genetic risk, they’re not to blame, they’ve discovered it, but already, the harm is there. But it’s better to take it into account, it means discovering it, they’re not the ones who are going to put it on, it’s the cause that’s there, it’s genetic, it means it exists and so much the better that I learn about it and if I and the members of the family have to be careful, they have to do several tests a year, a month... Why not?"*	Sara’s mother
*"Even though I said last time: we want to know... Yeah, up to a point maybe too."*	Lucien’s father

### Patients’ description and genetic or genomic testing context

The GeneInfoKid interview took place within a year of the cancer diagnosis for the vast majority of patients, with the remainder, now young adults, having been diagnosed up to 10 years. The patients’ mean age was 14.4 years (10–24); 7 patients had a haematological malignancy and 14 a solid tumour. Children and AYAs underwent somatic testing (*n* = 5), germline and somatic testing (*n* = 7), and germline testing (*n* = 9) including also for research purposes in four families (Table 4). How genetic testing was proposed varied in function of the hospital and the indication: one or more genetic consultations dedicated to genetic/genomic testing (*n* = 7); genetic information given during a standard oncology consultation preceded by genetic counselling (*n* = 7); genetic information given during an oncology consultation (*n* = 6); and no genetic consultation (did not attend or did not remember attending it (*n* = 1).

**Table 4:**
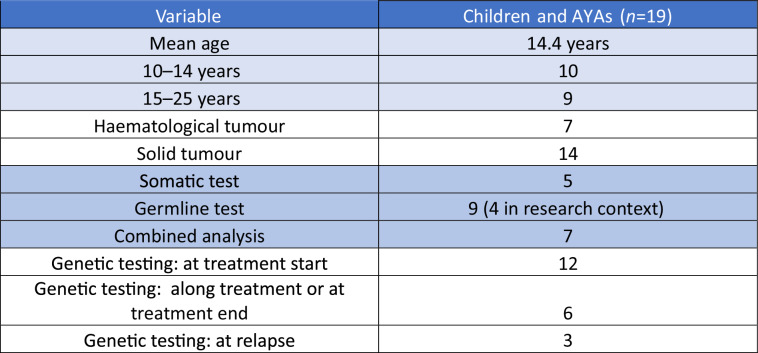
Patients*’* description and genetic or genomic testing context.

### Factors influencing genetic testing consent decision-making

The families’ feelings about consenting to genetic testing seemed to be influenced by how the test was proposed (i.e. consultation type, clinical and psychological context, perception of the type of test and medical indications).

#### Antagonistic feelings between genetic testing and cancer

Eight families described antagonistic feelings concerning genetic testing in the context of cancer. Cancer was described as a time of urgency and suspended present, due to the disease traumatic intrusion and death anxiety. It left little room to decide, *“it leaves no choice to decide, whatever it is”* [mother]. Some families explained their indifference and distance concerning genetic testing by its perceived lack of immediate usefulness for cancer treatment. Four families facing a therapeutic impasse initially showed few explicit expectations and distanced themselves from genetic testing. However, as the interview progressed, they expressed a form of last therapeutic hope concerning genetic testing. Four families would have liked to have more time to think about genetic testing to better understand what was done. Three parents would have preferred to wait until the treatment end.

#### Psychological availability and understanding

Most families showed only a relative psychological availability to genetic testing that influenced their understanding. As a protection when faced with too much information in a difficult context, some interviewees used some defence mechanisms. Four children and AYAs described this psychic unavailability: *“I was so down at that moment that even if I had been informed, it would have been useless. I wouldn’t have understood…”* [patient, 15–25 years]. This influenced the families’ capacity to consent. Some did not remember consenting to genetic testing, others vaguely, with a feeling of accumulating information *“without really digesting it”* [mother].

During the first interview before result, children and AYAs expressed their relative (6/14) or complete (5/14) lack of understanding of genetic testing. Four did not even know the word genetic. Two had forgotten they had a dedicated consultation (confirmed by the teams and parents). In the second interview after genetic test result, most children and AYAs (6/10) reported a lack of knowledge about genetic testing. One patient said that the adults had not told her about it. Five patients forgot about the result or most of the consultation or did not really understand the results and their implications. Only one patient (15–25 years) seemed to have generally understood the genetic results, but not the more complex information.

Before result one parent (1/13) reported a good understanding of genetic testing, four parents had a partial understanding, and two parents partially or completely forgot about it. After the results, most parents (7/11) described a form of psychological unavailability that may have hindered their understanding; two parents understood very little of the results, and two were completely unaware of the results. Two parents felt that they had understood the results and their implications. One of them said that the preparatory work (drawing the family tree) before the genetic consultation helped him to better understand the results. Two parents relied on their trust in medical professionals, “*I assume that they too are doing their best to find a cure for her. […] I don’t want to see it any other way […] it risks traumatizing my conscience (laughs)”* [mother]. The degree of understanding was also related to the initial expectations concerning genetic testing. When expectations were low (e.g. therapeutic impasse), families felt not implicated and did not try to understand.

#### Influence of the consultation type

In function of the genetic testing indication and hospital, information and/or the results were given in different contexts. The patients’ discourses suggested that these differences influenced their understanding and feelings about the received information.

In the first interview, parents who discussed about genetic testing (indication and results) during one or more dedicated onco-genetic consultation, better understood the involved issues and started to think about incidental findings (9/13). Conversely, parents who received genetic information during an oncology consultation (4/13) said that their attention was focused on their child’s cancer rather than on genetic investigations. Similarly, in the second interview, attention was more on the cancer than on the results when the genetic results were transmitted during an oncological consultation (4/11), as testified by a mother: *“in the end it almost… erased all this questioning about genetic predisposition”*. Genetic counselling helped some patients to better understand (3/14), but not others (4/14) due to concerns about their recovery, cognitive side effects, and young age. Children and AYAs who attended a onco-genetic consultation were more familiar with the meaning of the word genetics. Moreover, most parents and patients remembered having met a psychologist or psychiatrist during the cancer care pathway, but only two for a discussion on genetics.

#### Somatic/germline testing, a blurred perception by families

The understanding of the different test types by families (Table 1) was variable and some could not distinguish them (11/37). One parent who understood the difference between somatic and germline tests did not know exactly which test type was offered to his child. Three parents who understood that a tumour analysis was done to find therapeutic adjustments, expressed a sense of urgency that left little time to think about the other potential implications of results. Concerning testing done for research purposes (*n* = 4), one parent wondered about its temporality and the possible updating of his child’s consent when adult. One child felt that by entering a research protocol, he gave some sort of consent for germline testing. Two patients said that it was essential to participate in research but did not remember what the objective was.

### Is there room for an informed decision?

Some parents justified their consent by the healthcare professional’s explanation: search of the cancer cause (4/13), tumour characterisation and treatment optimisation (2/13), research purposes (2/13), and possible family prevention (2/13). A mother explained that the rarity of her child’s cancer led the healthcare professionals to propose genetic testing. Most children and AYAs (7/14) said that they were influenced by the adults, *“it was my parents who wanted to do it”* [patient, 10–14 years]. They remembered particularly the adults’ explanations on aetiological research and treatment. Before the test, decision-making varied. Among children and AYAs, some accepted without hesitation (2/14), while others (2/14) felt that they had not really decided, and they needed more explanations or time to form a more precise opinion (1/14). Some parents (3/13) accepted without hesitation and with a sense of urgency. Two parents expressed a sense of responsibility, or even a moral duty, to undergo genetic testing for the family, and thus the absence of a real choice, *“we don’t want anyone in our family to go through what we went through […] I say to myself, it’s our responsibility […] for our children, and our nephews and nieces”* [mother]. Two parents felt that they had not really consented, two forgot that they had given their consent, and another was uneasy about the test purpose.

### What place for children and AYAs?

When genetic testing was discussed and when the results were given, children and AYAs were sometimes not present. Some did not want or could not participate, but others did not know about it. A 16-year-old patient said, after some hesitation, that she would have liked to have been informed and present to talk about genetic testing. For others, discussion with their parents helped to better understand the issues.

In the first interview, most parents (7/13) said that they had discussed about genetic testing with their child to ensure that they were well informed and agreed to participate. Two parents did not discuss about genetic testing with their children and AYAs because they felt that they were psychologically unavailable. Four parents remembered that the healthcare professionals spoke directly to the children and AYAs and one parent emphasised the adjusted speech used: *“ she addressed him in very simple words. And… she let him talk a lot and rephrase what she was saying to make sure he understood”* [mother].

Concerning their child’s role during the result consultation, some parents remembered an active (2/11) or a more withdrawn presence (3/11). One parent noted that their child was not present at this consultation. Two parents said that they could not talk about genetic testing with their child because they were too preoccupied with the ongoing cancer treatment. One parent seemed to regret this: “*we quickly moved on to other things while, in fact, for her I think it was important and… I realize that we didn’t discuss it again*”. Two mothers remembered their child’s presence at the result consultation, whereas their children thought they were not there.

Thus, the children and AYAs’s place varied according to their age, their supposed maturity, the clinical and family situation, the parent-child relationship, and the place given by the healthcare professionals to them in this consultation.

### Anticipating and reacting to the genetic test outcome

#### Anticipating the results: expectations and fears

In the first interview, patients and parents expressed expectations and fears. For some children and AYAs, the lack of knowledge about genetic testing made it difficult to anticipate the results. They listed as expectations: aetiological explanation (4/14), altruistic participation in research (4/14), possible prevention (3/14), and therapy adjustment (3/14).

The parents’ expectations were: characterisation of their child’s disease (4/13), explanation of their child’s cancer aetiology (8/13), targeted treatment (8/13) (in line with the test aim), and hope of a last chance (2/13) in case of therapeutic impasse, and possible prevention (8/13) for their child and family. Five parents did not really know what to expect, and five expressed ambivalent expectations: anxiety about the possible impact of the results and reassurance about the possible prevention.

The fears expressed by the children and AYAs were: risk of relapse (3/14), risk of transmission to their future children (3/14), not knowing what to expect in terms of results (3/14), imaginary and erroneous representations of what genetics would allow (3/14) (e.g. to find an environmental cause of the cancer, to give access to one’s whole identity). Two patients feared that a non-response concerning their cancer aetiology with genetic testing would leave them with a void of meaning about the disease. A young patient evoked and denied the guilt her parents could feel, *“I’m not going to blame dad or mum, because they didn’t do it on purpose”* [patient, 10–14 years].

Parents feared the discovery of a familial risk (5/13), not understanding the results and their implications (2/14) and feeling some guilt (2/14) if the genetic result was positive. Two expressed concerns, about the future uses of their sample.

#### Reactions to the genetic testing outcome

In the second interview, patients and parents described different emotions and reactions to the result announcement. Some children and AYAs distanced themselves from the results (5/10) because they did not affect their daily life, unlike cancer. Three expressed relief when the results validated the therapeutic strategy, confirmed that the disease would not be transmitted to their future children, or confirmed the links of filiation. Two expressed disappointment or ambivalent feelings because the results did not elucidate the cancer aetiology.

Most parents experienced relief (9/11) because: the results validated the therapy or allowed a therapeutic adjustment (5/11), there was no family risk (3/11). Five parents were disappointed because: the results did not provide an aetiological explanation (4/11), lacked details and explanations (3/11) unlike their initial expectations, and did not allow treatment adjustment. Others expressed uncertainty (3/11), distance (3/11), or surprise (2/11) at the results. Two parents reported ambivalence between the non-hereditary transmission and the lack of an aetiological explanation.

For some children and AYAs and parents, genetic testing led to updating their personal history, the nature of the family ties, and the search of a disease explanation, *“I think that it is really the genetic test that has renewed um… the question… of the origin of the disease”* [patient, 15–25 years]. Some participants started to rethink about their initial cancer theories, especially because the negative test result left a void. After the genetic result consultation, three parents (3/11) and one child (1/10) remembered the cancer diagnosis announcement and compared their meaning and intensity. For one patient and her mother, the results gave a sense of possible empowerment, with cancer prevention measures to be put in place.

### Perception of incidental findings

The question “Did you expect other possible outcomes?” was not often asked, particularly to parents or patients with little knowledge about genetic testing. The concept of incidental findings was understood by one child (included in a research protocol), and by ten parents. For example, one mother (trio-based exome sequencing) heard that other discoveries were possible (predisposition to other cancers or diseases), but that such results would be given only if prevention was possible, which was acceptable for her. Three parents theoretically imagined the existence of incidental findings but did not relate them to their child. Six parents considered the discovery of incidental findings, which they called “predisposition” to other pathologies, for their child and/or the rest of the family. According to the information at our disposal, 14 children and AYAs (*n* = 7 germline testing and *n* = 7 mixed analysis) could have been concerned by a genomic technology with potential incidental data.

Participants expressed the desire to know about incidental findings to be better prepared, but also their fear. One mother said: *“Well, my fear is that, through the illness she already had, more serious things will be discovered behind it”*. After the genetic test result announcement, two parents expressed relief at the lack of incidental findings and one of them questioned the limit of his initial desire to know, *“Although I said last time: we want to know… Yeah, up to a certain point, maybe”*.

## Discussion

This study identified some subjective representations and perceptions of children and AYAs with cancer and their parents before and after genetic testing. The main emerging issues were the diversity of feelings, of understanding and consenting, their apprehensions and perceptions of the results, and finally their ambivalent attitude towards incidental findings.

Children and AYAs were more concerned about the risk of cancer relapse and transmission to their offspring, and lack of answer on their cancer aetiology. Conversely, parents feared the family risk. Patients and parents talked about the possible feeling of guilt of the parents about family transmission. Healthcare teams should consider these representations because they influence the families’ listening and psychological availability [19, 20].

The perception of consenting was sometimes blurred. Some families expressed the fact that they did not feel they had had the choice of whether to undergo genetic testing, but that they trusted the health professionals in any case. This raises questions about the genetic testing consent validity, which is required by the French law (article 16 of the French Civil Code). Consent may have different value, such a symbolic one, and may be expected as a process for patients and their parents. We previously proposed that the delivery of results which does not concern the child’s treatment and direct care could entail a renewed consent to preserve the right not to know, but also to change one’s mind [20]. Discussions at a distance from the genetic proposal and urgent care, or even at the time of the child’s transition to adulthood, also should be considered [20, 21]. Therefore, it might be important to create spaces to talk again about what the family has agreed to and its implications during cancer treatment, remission, at the child’s age of majority, and even after the child’s death.

The understanding of the genetic proposition varied among parents and children and AYAs and according to the context, raising questions on how to better deliver information. The traditional onco-genetic consultation increases understanding [17, 22, 23]. For genomic proposition, other strategies have been experienced such as the two-visit consent model [24] or a systematic consultation with a genetic counsellor at the cancer treatment initiation [25]. For example, the construction of the family tree allows a first representation and understanding of the personal and family issues linked to a future genetic test. The timeframe of information transmission also could be improved. One parent suggested to give a document summarising the ongoing genetic testing process. Patients’ understanding was much poorer, and not only because of their age. This suggests that information for children and AYAs should be adapted to what they can and wish to hear, in a dynamic interchange, and with suitable supports [21, 26]. As psychological unavailability and forgetfulness could be protective mechanisms in children and AYAs with cancer and their parents, it may be necessary to give professionals the means to identify them. A psychologist consultation could be systematically proposed to identify the patients’ psychological readiness and to adapt the genomic pathway. Psychologists also can play an essential role in supporting families by discussing with them their representations, expectations, and emotions before and after the results [22, 23].

Some emotions and reactions to the result announcement were similar in children and AYAs and parents: relief, disappointment (due to the lack of aetiological explanation), distancing, low emotional distress, ambivalence. Conversely, others were specific to parents, such as uncertainty and surprise. The low distress level associated with the results could be linked to the cancer experience that overshadowed all other fears, as previously reported [3, 26]. These differences between children and AYAs and parents were previously described [3, 27] and highlight different expectations (altruism is more emphasised by adolescents, while hope for a cure is stronger among parents).

Lastly, it was often difficult to discuss about incidental findings because of the interviewees’ limited understanding of genetics. Parents with a relatively accurate understanding said that they wanted to know but were also afraid [3]. A recent study highlighted the parents’ strong expectations regarding updating the genomic results for their children when new information becomes available, but not after the child’s death for some of them [28].

## Study limitations

As the study population was heterogeneous in terms of disease, treatment, prognosis and genomic test type (somatic or constitutional), the psychological issues experienced by children, AYAs and parents were different. Moreover, professionals involved were not the same in the different teams, with or without geneticist, genetic counsellor, or psychologist. We can consider that we have reached data saturation for most of the topics covered. However, we have not yet reached this criterion for some of them, particularly those relating to children and AYAs’ perspectives. As this study was entirely exploratory, we did not collect information on the parents’ socio-educational level, nor on the patient’s medical history, and this may perhaps limit certain interpretations of the responses.

## Conclusion

Nevertheless, the qualitative approach enriched the understanding of the various and complex situations in real-life clinical situations. In conclusion, the interviewed families expressed varying levels of understanding and different apprehensions concerning genetic testing. Some families would have liked to have more time to think about the test meaning or at a distance from the acute phase. Therefore, it should be important to develop an extended time and step-by-step approach to explain genetic or genomic testing during dedicated multidisciplinary consultations or during the paediatric oncologic consultations with other healthcare professionals (genetic counsellor, psychologist).

The themes identified by the qualitative analysis will allow us to design specific questionnaires to be tested in larger populations in the quantitative study of the GeneInfoKid project. This will provide more information to understand the families’ expectations and needs, particularly on incidental findings, and to promote shared decision-making by healthcare professionals, parents, and children and AYAs.

## Supplementary information

Semi-structured Interview grids

## Data Availability

The data that support the findings of this study are available from the corresponding author upon reasonable request.
