# Supplementary material for: Psychological and ethical issues raised by genomic in paediatric care pathway, a qualitative analysis with parents and childhood cancer patients
Source: Eur J Hum Genet. 2024 Jul 13;32(11):1446–55. doi: 10.1038/s41431-024-01653-4 (PMC11577070; doi:10.1038/s41431-024-01653-4)
Supplement: Supplementary file 1 — Semi-structured Interview grids [file 41431_2024_1653_MOESM1_ESM.pdf]

Supplementary Information

1- Semi-structured Interview grid for children, adolescents and young adults

NB: the questions should be formulated using vocabulary and a style appropriate to the child's age.

<u>At the time of the test</u> :

Short presentation of the study: the aim of this study is to identify the ethical, legal, psychological and informational issues surrounding the incidental data that may appear during a new-generation genetic test for childhood cancer.

- *Psychological objectives: to gain a better understanding of the psychological issues for patients and parents of patients, in order to adjust genetic consultations and propose recommendations for family support.*
- *The value of studying the experiences of children and adolescents and young adult with genetic testing: it is through what you tell us about your experience that we will be able to better understand the issues you are going through and propose adjustments to your care.*

During an interview lasting around 30 minutes, we will talk about your experience as a child or young adult who is/was ill, and in particular about the genetic tests you have been offered, and what you think and fear about them. I'm going to ask you some questions, which you are free to answer or not. There are obviously no right or wrong answers, what we're really interested in is hearing your opinion and your feelings.

- ❖ Current context:
  - Family: current family configuration (parents together, separated, siblings, etc.) and current family organisation in the context of the illness.
  - The illness: the psychologist will ask the parents to talk briefly about their child's illness, and to specify the current situation in the child's life *(in particular, try to hear about any current traumas).*

- ❖ Reformulation of the benefits of genetic analysis
  - Has the child already had experience/knowledge of genetics elsewhere
  - The psychologist will ask the child/young adult to explain in his or her own words the advantages of genetic testing as he or she sees them.

- ❖ Expectations of the primary result, different types of result? What are their expectations?

The following hypotheses will be explored (without being formulated verbally by the psychologist)

- *Use for therapeutic or family prevention purposes?*
- *Giving meaning to the illness?*
- *Clearly expressed therapeutic hope associated with the approach, or simple acceptance of medical advice?*

- ❖ Fears associated with the primary outcome:
  - *Guilt about worrying parents (perhaps one day passing on the disease?)*

- *Risk to siblings?*
- *Do you have any other fears apart from the medical benefit sought? (An announcement that would have symbolic effects on filiation and transmission? ...)*

❖ Attitudes regarding incidental results

- Understanding of this possibility

Do children/adolescents/young adults understand the difference between a primary result and an incidental result?

- Need for further explanation? Referral to whom?

❖ Decision-making process

The following hypotheses will be explored (without being formulated verbally by the psychologist)

- *What decision: to know or not to know? Family discussion?*
- *Acceptance of the proposal for genetic analysis without any real hope? "Moral obligation to know?*
- *Ambivalence?*
- *Discovery of a new risk → increased fears for the future?*
- *Fear of uncertainty linked to the result?*
- *Possibility of projecting oneself into a more distant future when a future risk is mentioned? Can the child talk about risks that concern adulthood?*
- *Can they offer prevention to their family? Do they want to protect their brothers and sisters with this type of test?*

In conclusion, do you have any comments, remarks or additional questions?

<u>At the time of the result</u> :

- *Collection of current context (if patient not met in pre-test):*
  - *Family: current family configuration (parents together, separated, siblings, etc.) and current family organisation in the context of the illness.*
  - *The illness: the psychologist will ask the parents to talk briefly about their child's illness, and to specify the current situation in the child's life (in particular, try to hear about any traumatic events).*

- The psychologist will have the results formulated and will ensure that the child understands them. If the results do not seem clear to them, do they know who to contact?

- The various emotional reactions will be noted *(tones of anxiety, astonishment, etc.)*.

- The psychologist will assess
  - Whether the result matches the patient's expectations. If the results do not seem clear to them, do they know whom to contact?
  - Their perception of the consequences and possible implications for their treatment and beyond.
- Where appropriate, the psychologist will explore reactions to any secondary results:
  - The child's emotional reactions will be noted.
  - Is the child able to project the medical consequences in terms of prevention?
  - Does this symbolically change things for them in their family history? And in their projections for the future more generally?
- Caregiver-patient relationship:
  - What did you think of the timeline in which you were offered the genetic tests? What would have been a better timeline for you?
  - Did you meet with a psychologist or psychiatrist during the genetic process? If so, how did this come about, on whose initiative and where? What did you think, was it useful and was it the right time? If you didn't meet one, were you offered one, and if not, did you miss it?
  - In your opinion, what could have been done differently to help you better understand the issues involved in these genetic tests, to make a decision more easily and to cope better with the results? And what did you find particularly helpful that was already in place?

To conclude, do you have any comments, remarks or additional questions?

2 - Semi-structured interview grid for parents

<u>At the time of the test</u> :

Short presentation of the study: the aim of this study is to identify the ethical, legal, psychological and informational issues surrounding the incidental data that may appear during a new-generation genetic test for childhood cancer.

- *Psychological objectives: to gain a better understanding of the psychological issues for patients and parents of patients, in order to adjust genetic consultations and propose recommendations for family support.*
- *The value of studying parents' experiences of genetic testing: it is through what you tell us about your experiences that we will be able to better understand the issues you are going through and propose adjustments to your overall care.*

In an interview lasting around 30 minutes, we will talk about your experience and your experience as a parent of your child's cancer journey, and particularly of the genetic tests you have been offered, and what you think and feel about them. I'm going to ask you some open-ended questions, which you are free to answer or not. There are obviously no right or wrong answers, what we're really interested in is hearing your opinion and your feelings.

- ❖ Current context:
    - Family: current family configuration (parents together, separated, siblings, etc.) and current family organisation in the context of the illness, particularly in the parental couple. (Who is the parent we are meeting in relation to this family organisation?)
    - The illness: the psychologist will ask the parents to talk briefly about their child's illness, and to specify the current situation in the child's life (in particular, try to hear about any traumatic events).

- ❖ Reformulation of the benefits of genetic analysis
    - Have they already had experience/knowledge of genetics elsewhere?
    - The psychologist will ask the parents to explain in their own words the benefits of genetic testing as they see them, in the specific case of their child.

- ❖ Expectations of the primary result, different types of result?

What are their expectations?

The following hypotheses will be explored (without being formulated verbally by the psychologist):

- *Use for therapeutic purposes, or for family prevention (will this result be of practical use in the therapeutic management of their sick child, and/or for the prevention of other children and the parents themselves)?*
- *Giving meaning to the disease. Will the genetic result help us to understand the origins of the disease? Is the explanation provided by the test result associated with an idea of relief, or on the contrary with the notion of seriousness?*
- *Is the therapeutic hope associated with the test clearly expressed, or is it simply acceptance of medical advice?*

- ❖ Fears associated with the primary result:

The following hypotheses will be explored (without being formulated verbally by the psychologist):

- *Guilt: if a genetic predisposition is diagnosed, and possibly passed on by one of the parents, is there an associated feeling of guilt?*
- *Risk for other children: similarly, if a genetic predisposition is found, will this induce a fear or threat for other children?*
- *Do you have any other fears beyond the medical benefit sought (announcement that would have symbolic effects on filiation and transmission, etc.)?*

(Include personal beliefs and representations about genetics)

- Attitudes towards incidental results
    - *Understanding of the possibility of incidental results*
    - *Do parents know the difference between a primary result and an incidental result?*
    - *Do they need any further explanation at this stage? Do they know who to contact if this is the case?*

- Decision-making process

The following hypotheses will be explored (without being formulated verbally by the psychologist):

- Did the parents decide to find out the incidental results? If yes, why? If not, why not? Was this discussed within the family? With whom? What freedom do they feel they had in this process? What factors influenced the decision? Do they feel they had enough information to make a decision?
- What consequences and implications do they imagine these secondary results will have? Does the discovery of a new risk increase their fears for the future? Or, on the contrary, do parents emphasise the possibility of preventing this new risk if detected early?
- Do they accept the offer of genetic analysis without any real hope? "Moral obligation to know?
- Fear of the uncertainty linked to the result?
- Ambivalence?

To conclude, do you have any comments, remarks or additional questions about this interview?

At the time of the result :

- *Collection of current context (if parent not met in pre-test):*
  - *Family: current family configuration (parents together, separated, siblings, etc.) and current family organisation in the context of the illness, particularly in the parental couple. (Who is the parent we are meeting in relation to this family organisation?)*
  - *The illness: the psychologist will ask the parents to talk briefly about their child's illness, and to specify the current situation in the child's life (in particular, try to hear about any traumatic events).*

- The psychologist will have the results formulated and will ensure that the parents understand them. If the results do not seem clear to them, do they know who to contact?

- The various emotional reactions will be noted (what tones of anxiety, shock, etc.).

- The psychologist will assess
  - The match between the expectation and the result: does the result match the parents' expectations? What do these matches or variations produce in the parent?
  - Their perception of the consequences and possible implications for their child's treatment and beyond.

- Where appropriate, the psychologist will explore reactions to any secondary results:
  - In the same way, the parents' emotional reaction will be noted.
  - Are the parents able to project the medical consequences in terms of prevention?
  - Does this symbolically change things for them in their family history? In their projections for the future more generally?
  - Does it change things in their relationship with their sick child, and with their other children?

- The relationship between carers and patients:
  - How did the carers on the genetic team explain the genetic test protocol to you, from the time you were informed to the time you told your child?
  - What did you think of the chronology in which you were offered the genetic tests? What do you think would have been a better timeline?
  - Did you meet with a psychologist or psychiatrist during this genetic process? If so, how did this come about, on whose initiative and where? What did you think, was it useful and was it the right time? If you didn't meet one, was it suggested to you and if not, did you miss it?
  - What do you think could have been done differently to help you better understand the issues involved in these genetic tests, to make a decision more easily and to cope better with the results? And what did you find particularly helpful that was already in place?

What do you think the other parent would think of your answers? Would they agree or disagree on one or more points?

Finally, do you have any comments, remarks or additional questions?
